# A new systematic collection and classification of odour words by using a product review dataset

**DOI:** 10.1371/journal.pone.0289368

**Published:** 2023-08-10

**Authors:** Naoya Zushi, Genki Takeuchi, Midori Ogawa, Naomi Gotow, Hideki Kakeya, Tatsu Koabayakawa, Saho Ayabe-Kanamura

**Affiliations:** 1 Graduate School of Comprehensive Human Sciences, University of Tsukuba, Tsukuba, Ibaraki, Japan; 2 Graduate School of Science and Technology, University of Tsukuba, Tsukuba, Ibaraki, Japan; 3 Institute of Human Sciences, University of Tsukuba, Tsukuba, Ibaraki, Japan; 4 Human Informatics and Interaction Research Institute, National Institute of Advanced Industrial Science and Technology, Tsukuba, Ibaraki, Japan; 5 Institute of Engineering, Information and Systems, University of Tsukuba, Tsukuba, Ibaraki, Japan; Federal University of Paraiba, BRAZIL

## Abstract

The odours encountered on a daily basis are dependent on an individual’s society and culture. Therefore, when conducting olfactory tests, the odour stimuli utilized must be appropriate for the individual’s environment. In this study, we gathered and classified the odours experienced by Japanese individuals in their daily lives through a large dataset of product reviews encompassing food and household items. Specifically, we performed morphological analysis on product review sentences in Japanese that contained odour descriptions, and we compiled the nouns used to describe odours. A total of 617,208 sentences that reviewed odour experiences and their corresponding nouns were collected. The top 100 frequently used odour nouns were classified into 15 clusters according to the context in which they were used. The methodology employed in collecting and classifying odour nouns as presented in this study can be utilized in other situations. It can assist in selecting appropriate odour stimuli for the olfactory test based on the society, culture, and time period.

## Introduction

Several olfactory tests have been developed in the past, including the University of Pennsylvania Smell Identification Test (UPSIT) [1] and Sniffin’ Sticks [2, 3]. It is feasible to establish a standardized test method globally to measure human olfactory ability to detect a specific odorant. However, as the odour perceived is reliant on social and cultural backgrounds [4–7], certain aspects of tests assessing the ability to identify odours cannot be addressed by universal standards. The UPSIT, developed in the USA, is a widely recognized olfactory test among researchers globally, but its stimuli contain odours unfamiliar to Japanese and European individuals. As a result, the 12-item Cross-Cultural Smell Identification Test (CC-SIT) was developed as a revised version, replacing some odours with those that are more familiar to an individual’s experiences and culture [8]. Similarly, a revised version of Sniffin’ Sticks, called the Chinese Odor Identification Test (COIT), has been developed, as some of the odors used in Sniffin’ Sticks, developed in Germany, are unfamiliar to individuals residing in China [9].

Approximately 400 olfactory receptor-expressing genes have been identified in humans [10], and it has been shown that a single odourant can activate multiple receptors [11]. Furthermore, most of the odours we perceive are complex odours composed of multiple olfactory compounds. Given this neural basis of olfaction, measuring the ability to detect specific odourant compounds does not necessarily indicate whether our olfaction is functioning normally in daily life. Identifying an olfactory stimulus as an odour mixture that we are exposed to in our daily lives is the most appropriate indicator to investigate the relationship between the olfactory system and neurological diseases, as it requires recognition based on the memory of previously experienced odours. Currently, the T&T olfactometry [12] is used as an olfactory test in clinical settings in Japan, but the olfactory stimuli used in this test are all single chemical substances and may not reflect the olfactory abilities required on a daily basis. Like the UPSIT, Sniffin’ Sticks, CC-SIT and COIT, the Odor Stick Identification Test for Japanese (OSIT-J) is a method for testing the ability to identify the odour [13, 14]. This test has been found to be suitable for measuring general olfactory ability in clinical situations and olfactory studies in otolaryngology and neurology [15–18]. The development of OSIT-J began with a classification of daily odours in Japanese individuals [19]. The odour descriptors used for this classification were based on free-text data obtained for various olfactory stimuli in several previous experimental studies. Participants were asked to classify the odours based on the similarity of the impressions elicited by these odour descriptors. From this classification, representative odours were selected for each cluster and reproduced as olfactory stimuli. OSIT-J scores based on the daily odours of the Japanese have been found to be significantly lower in the US individuals than in the Japanese individuals [20], suggesting that the appropriate olfactory test depends on the odours to which individuals are exposed daily.

Participants in the OSIT-J odour selection process were selected from a limited number of regions [19]. However, it is unclear whether data from this limited area can be considered representative of the country as a whole. Even within the same Japanese environment, the globalisation of food and the diversification of lifestyles in recent years have led to the experience of new odours that were not present in the average Japanese lifestyle in the past. In addition, while the positive effects of olfactory stimulation such as aromatherapy have been demonstrated [21, 22], the health and psychological damage caused by using excessively perfumed products has also become a social problem [23, 24]. Therefore, it is necessary to conduct an extensive survey of the odours that the modern Japanese is frequently exposed to in their daily lives, to re-establish appropriate olfactory stimuli for the olfactory identification ability test for them, and to establish a method for the continuous investigation of the ever-changing daily odours in the future.

The internet can be seen as an effective way of collecting data on people’s current daily odour experiences across Japan, rather than in specific areas. Here, the problem is that there is no odour-specific vocabulary to describe odour quality when collecting data. For example, in the case of vision and taste, we can express the qualitative experience of perception by means of modality-specific vocabularies, such as sweet, salty, sour, bitter and umami in the case of taste, or red, blue and yellow in the case of vision. However, this is not the case for olfactory experiences, so when we describe an odour, we often express its quality by associating it with concrete objects, such as "the flavour of roses" or "the scent like roses". Indeed, during the development of OSIT-J, the classification of odours was carried out using concrete nouns [19]. Therefore, we devised a method to collect descriptions of odour experiences using concrete objects from texts posted on the Internet. In addition, we considered that the contextual information of the olfactory experience could be taken into account by co-occurring words used in the sentences, and the distance between odour words could be determined. The odour stimuli used in the olfactory test should be as few numbers as possible and selected from odours experienced in different daily situations, taking into account the simplicity and effectiveness of the test. Hence, this study first collected words describing familiar odours in the lives of modern Japanese from a large dataset of product reviews posted on the internet. Then, 100 frequently experienced odours were selected and hierarchically clustered according to the context in which they were experienced. In addition, based on these data, we propose an appropriate and sustainable method for selecting suitable odours as stimuli for olfactory tests for modern Japanese.

## Methods

### Dataset

We utilized the product review dataset from the "Rakuten Dataset" available on the IDR Dataset Service of the National Institute of Informatics, Japan (https://rit.rakuten.com/data_release/). This comprises product reviews posted on Rakuten Ichiba, a Japanese e-commerce platform offering a diverse range of products such as food, beverages, electronics, home furnishings, pharmaceuticals, books, cosmetics, and pet products from January 2015 to December 2019. The dataset encompasses a total of 69,624,575 reviews written in Japanese.

### Data processing

**Fig 1:**
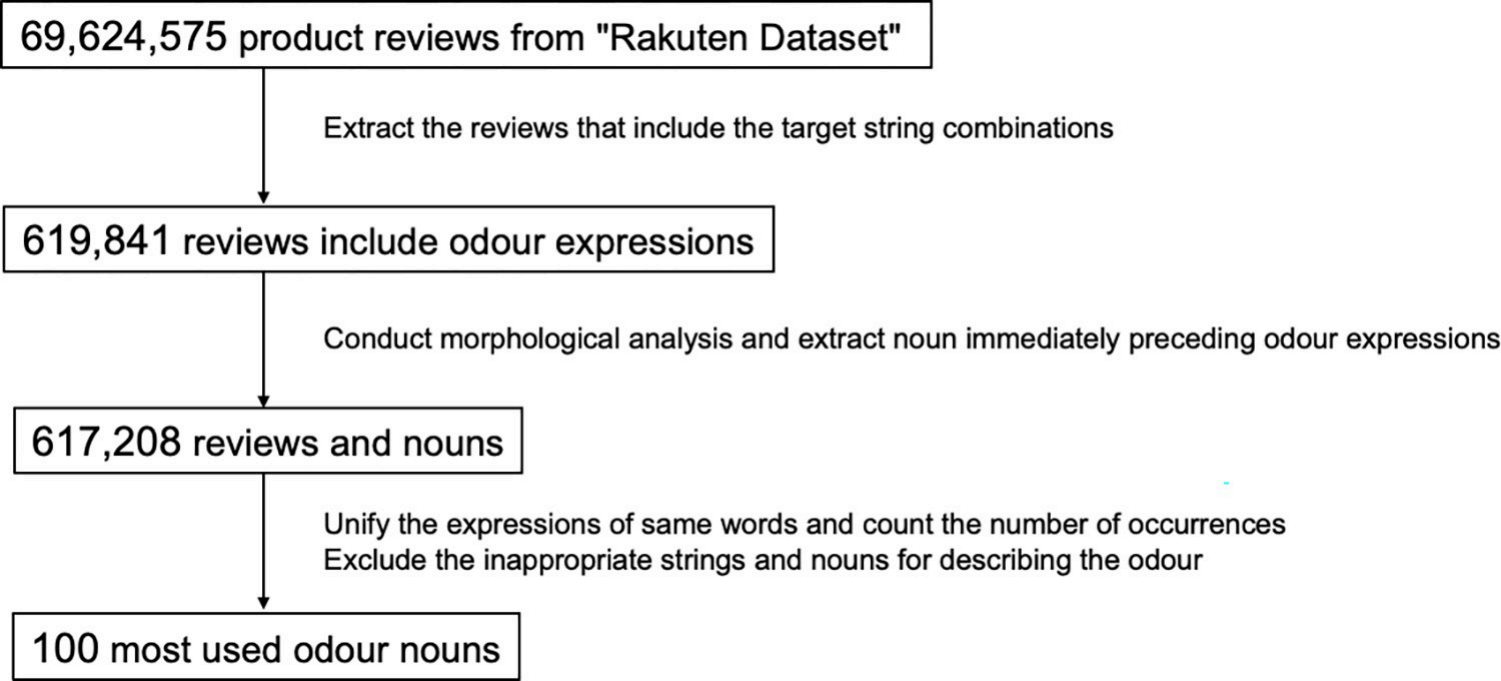
Overview of the data processing flow. All processes were conducted in Japanese.

The dataset was processed using Python v3 (Fig 1). From the product review data, a total of 619,841 reviews containing the target string combination "no/noyouna/mitaina"+"nioi/kaori" as odour expressions were extracted. "X no nioi/kaori" is equivalent to "odour of X", "X noyouna nioi/kaori" and "X mitaina nioi/kaori" equivalent to "odour like X". This method was employed as expressions such as "X no nioi" in Japanese are indicative of a word describing an odour, with the noun (X) preceding the particle "no" considered as such. The Japanese language has ideographic characters (Chinese characters) and phonetic characters (Katakana and Hiragana), and different strings can have the same meaning. There are several strings representing "nioi" (匂い/臭い/ニオイ/におい) and "kaori" (香り/薫り/かおり), but they were all treated in the same way as strings of odor expressions.

**Fig 2:**
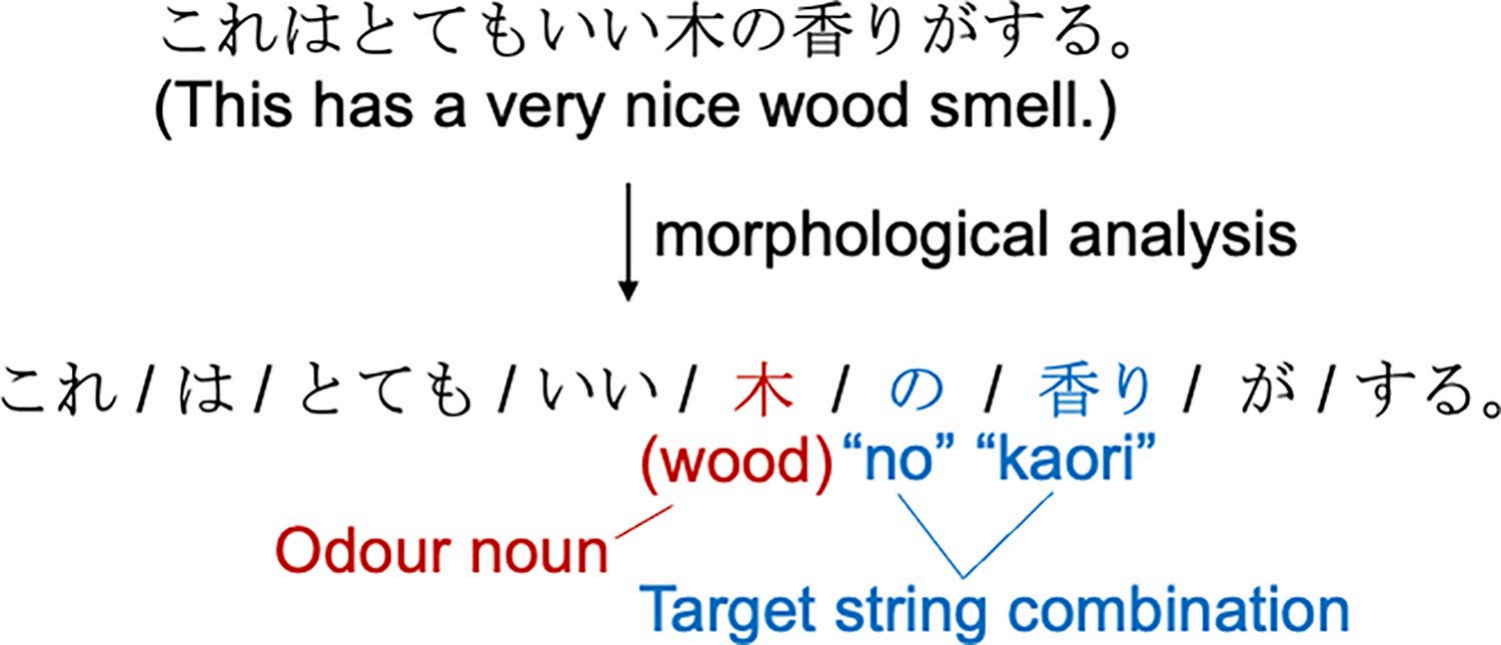
How to extract the odour noun from the review sentence. In this study, the target string combinations were "no/noyouna/mitaina"+"nioi/kaori." In Japanese, the expression "X no nioi/kaori" denotes the odour of X itself, whereas "X noyouna nioi/kaori" and "X mitaina nioi/kaori" are used to express the nuance of smelling like X. Therefore, the sentences were segmented into words, and nouns appearing in X were extracted as odour nouns.

**Fig 3:**
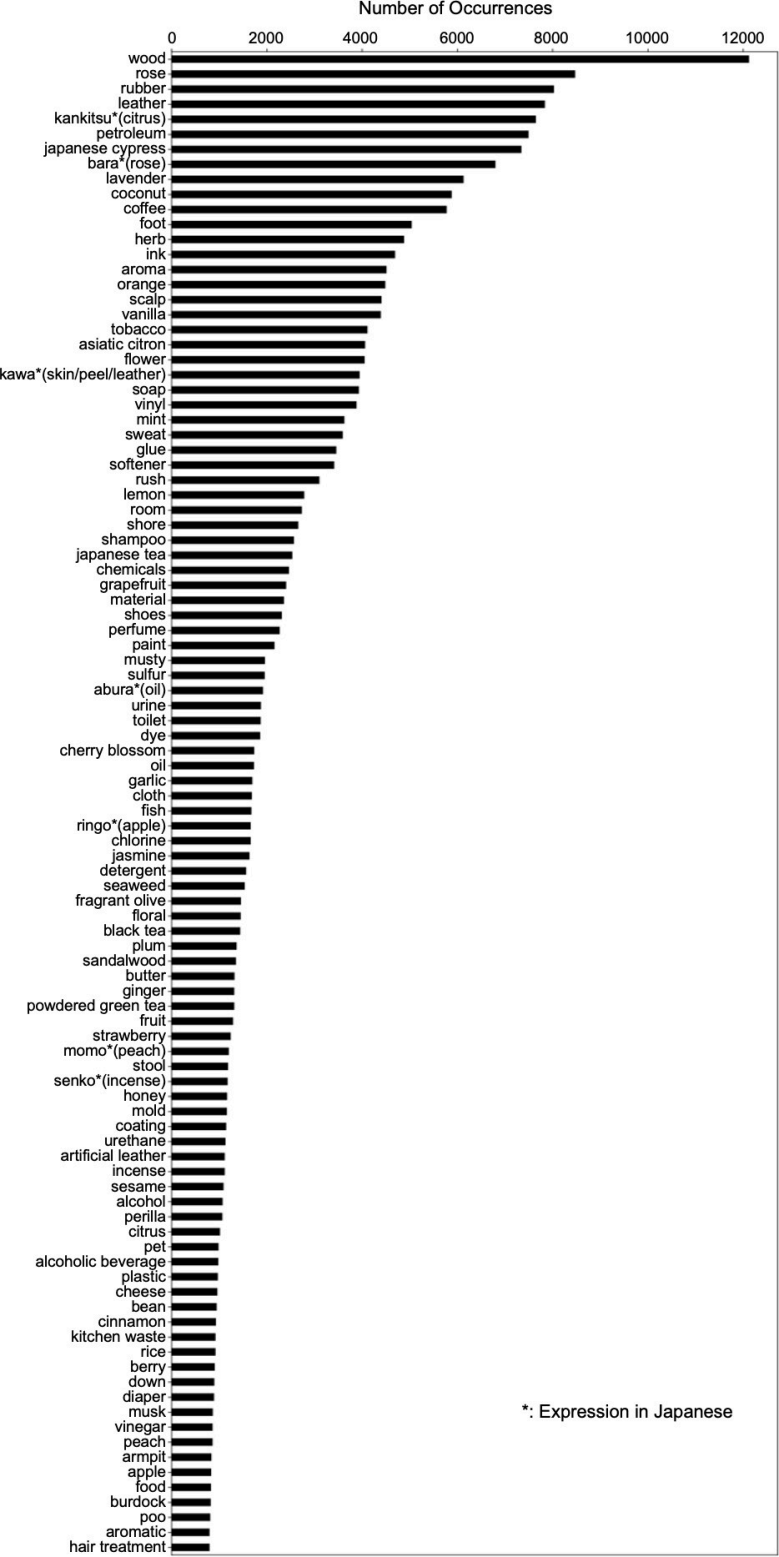
The 100 most used odour nouns and their number of occurrences. All words were collected in Japanese.

The extracted review data were then analyzed using the Japanese morphological analysis software MeCab [25] and its system dictionary mecab-ipadic-NEolog [26] to extract the nouns immediately preceding the target string combination (Fig 2). The mecab-ipadic-NEolog system dictionary can handle words and phrases that are not properly segmented by the standard MeCab dictionary by adding new specific expressions obtained from linguistic resources on the Internet. For example, if the noun "coconut oil" appears before the target string combination, the standard MeCab dictionary splits it into "coconut" and "oil" and cannot extract it correctly as a single noun. In this case, "oil" is incorrectly extracted, but mecab-ipadic-NEolog can be used to correctly extract "coconut oil". As the dataset was constructed from reviews posted on the Internet, analysis using this dictionary was considered appropriate. As a result, a total of 617,208 nouns and reviews containing them were identified. The same words written in different forms (Chinese character/Katakana/Hiragana, full width/half width) were then unified into the form with the highest number of occurrences and the occurrences of each word were counted. After excluding 27 strings that were either not nouns or considered inappropriate as odour nouns (S1 Table), the 100 most frequently used odour nouns were selected for further analysis (Fig 3). The exclusion process was carried out by excluding words that six human evaluators agreed did not describe a concrete object that emitted an odour, until all the top 100 nouns were concrete objects.

Hierarchical cluster analysis was conducted on the selected 100 odour nouns extracted by calculating the distance between nouns based on their co-occurrence frequency in the review. This analysis was performed based on the Euclidean distance between nouns using Ward’s method [27]. The Euclidean distance was calculated by normalising the feature vector of co-occurrence frequencies with other nouns in the review so that the sum of each vectors equals 1. For example, when taking the noun "eau de parfum" as an example of a co-occurrence frequency vector, the value of this vector is large (0.000318) in the noun "perfume", because "perfume" and "eau de parfum" co-occur frequently in the same review. This vector is also large in "vanilla" (0.000253), which is often used as a scent for perfume, but the noun " kitchen waste" and "eau de parfum" occur less frequently in the same sentence, so this vector in " kitchen waste" is small (0.000125). Therefore, the difference in the size of the vector "eau de parfum" in each noun is smaller for "perfume" and "vanilla" (0.000065) than for "perfume" and " kitchen waste" (0.000193). In this study, the co-occurrence frequencies of all nouns in all reviews are treated as vectors, and the Euclidean distances between nouns are determined from the difference in their magnitudes, thus clustering them to reflect the context in which the odour was experienced. The sum of each vector was normalised to 1 to remove the bias introduced by the vectors of some frequently occurring nouns. To determine the appropriate number of clusters, the Davies Bouldin Index [28] was calculated. For the hierarchical cluster analysis and the calculation of the Davies Bouldin index, "scipy.cluster " module and "sklearn.metrics" module were used in Python v3.

## Results

**Fig 4:**
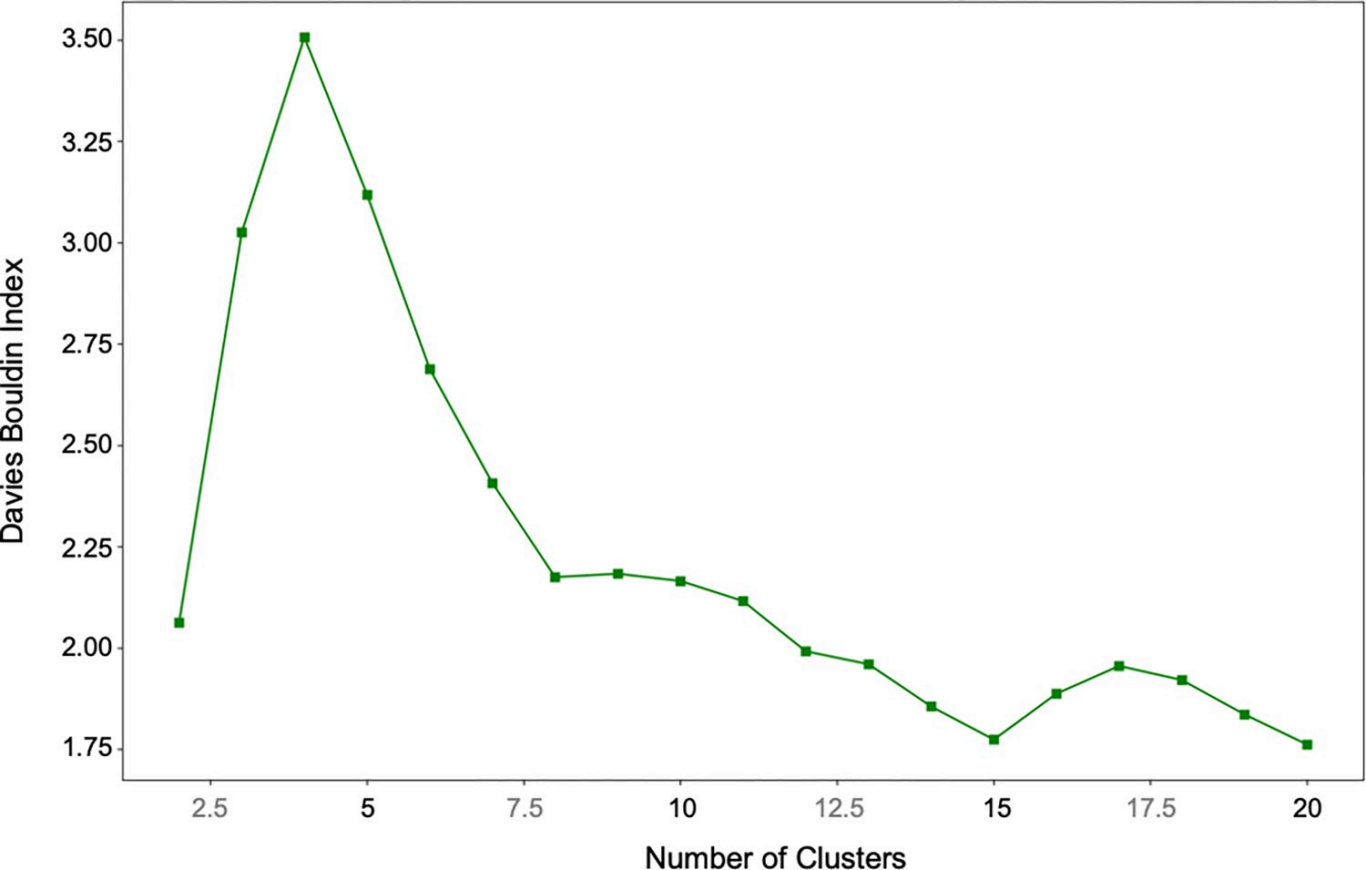
Davies Bouldin index by the number of clusters.

**Fig 5:**
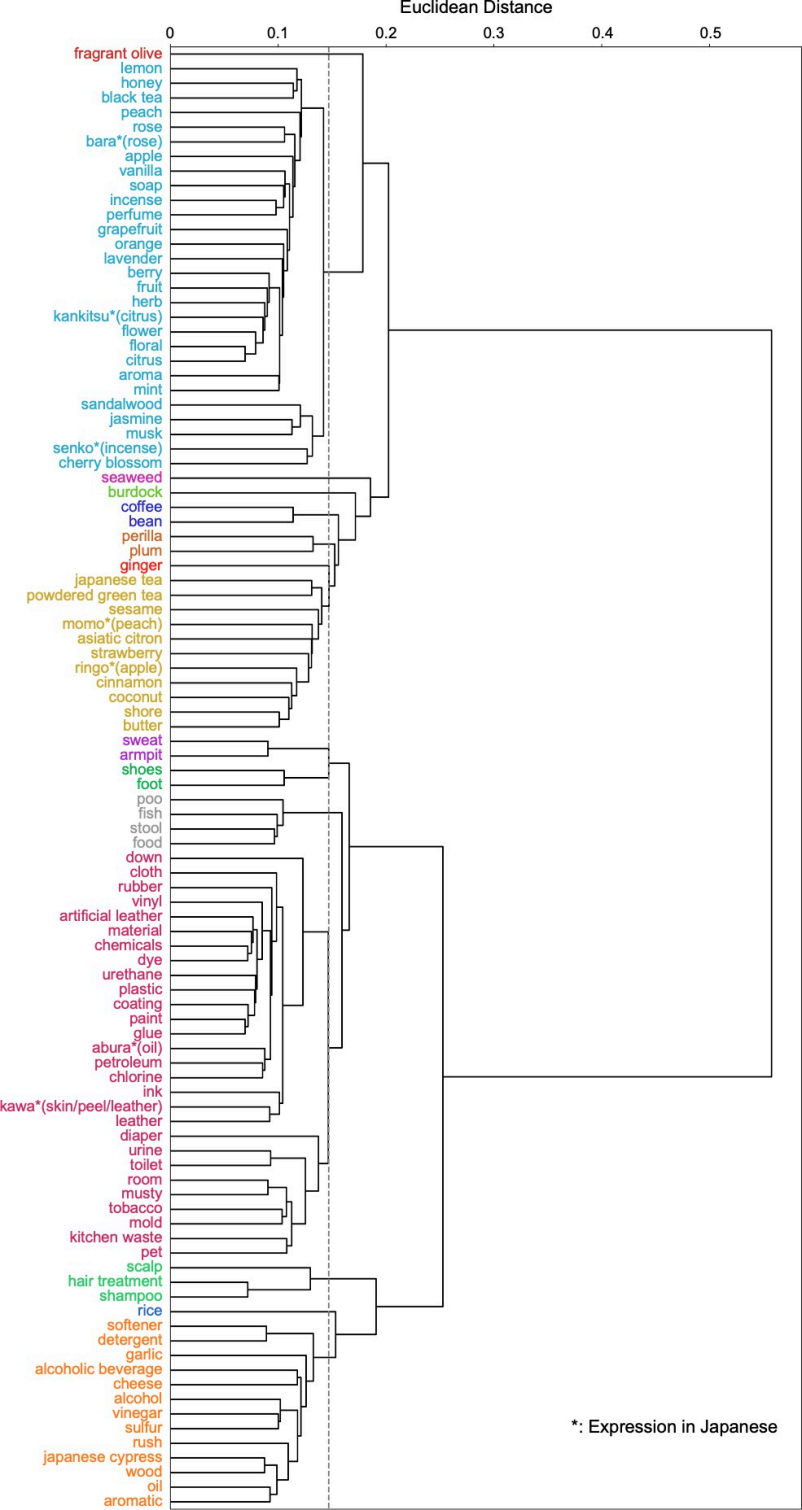
Hierarchical clustering dendrogram using Euclidean distance and the Ward method. These were colour coded into 15 clusters based on the Davies Bouldin index. The dotted line indicates the threshold for clustering, which is approximately 0.147.

With reference to the Davies Bouldin index for the obtained distance matrix (Fig 4), a hierarchical clustering structure with 15 clusters is shown in Fig 5.

## Discussion

In this study, we collected and classified the odours experienced by modern Japanese in their daily lives, based on 619,841 review data extracted from a large dataset. This dataset consisted of product reviews posted online from across Japan between January 2015 and December 2019. It is important to note that these reviewers were not deliberately asked to describe odour experiences. The odours used in traditional olfactory tests were selected as a result of the intentional recognition of odours by researchers and experimental participants [1–3, 8, 9, 13, 14]. Moreover, previous studies on the classification of odours have been criticized for not taking into account the target object of the classification or for focusing only on specific categories of odours [29]. However, the e-commerce website that was the source of the dataset used in this study did not provide any inducement in terms of odour experiences when submitting reviews, and the odour words expressed were natural expressions of individuals’ experiences. In this respect, we believe that we were able to collect more daily odours than with traditional methods. As a result, the final selection of the 100 most used odour nouns encompassed a broad range of odours, from pleasant to unpleasant, that are typically encountered in various contexts of daily life.

Among the 100 odour nouns selected here, there are some that are conceptually related, such as "flower" and "(rose, lavender)", or "fruit" and "(orange, apple)", where the former encompasses the latter. Although these words originally represent different levels of conceptual information, each word was chosen to represent the odours that individuals perceive in their daily lives. Therefore, in this study, they were treated equally as odour nouns in terms of classifying the olfactory experience in daily life. Similarly, words that have the same meaning when translated, such as "rose" and the Japanese expression "bara" or "peach" and the Japanese expression "momo", were treated as independent odour nouns, based on the interpretation that different words were chosen to express the perceived olfactory experience. Hierarchical clustering was performed on the 100 odour nouns thus selected. This clustering can contribute to the classification of selected odours experienced in different contexts and to the selection of stimuli for the olfactory test. Currently, the T&T olfactometry [12], an olfactory test using single chemical substances, is covered by insurance in Japanese otorhinolaryngology. However, given the neural basis of olfaction, measuring the ability to detect a specific odourant does not measure whether the individual’s olfaction is functioning adequately in daily life. It is therefore appropriate to use odours that individuals experience daily as stimuli for tests that measure olfactory ability needed in daily life. For example, the most frequently used odours within each of the 15 clusters obtained in this study can be selected ("fragrant olive", "rose", "seaweed", "burdock", "coffee", "plum", "ginger", "coconut", "sweat”, “foot", "fish", "rubber", "scalp", "rice", and "wood"). We believe that an olfactory test using stimuli that reproduce these odours would be more suitable for measuring the olfactory abilities that modern Japanese need in their daily lives because these odours are experienced in many daily situations and each odour has different characteristics.

Many previous studies have conducted perception-based odour classification [29, 30]. These were classified odours based on odour descriptor evaluations [5, 31, 32], similarity evaluations [19, 33, 34] and a database of quantitative odour profiles [35–37]. A perception-based odour classification is crucial in understanding the biological and functional properties of human olfactory perception. In contrast, in this study, the classification was based on the context in which the odour was experienced, referring to other co-occurring words expressed in the review sentences. The significance of perception-based and context-based odour classification differs, and the latter may be more appropriate for selecting stimuli for the olfactory test to measure the olfactory ability required in daily life.

A limitation of this study is that the dataset was derived from product reviews, which encompassed a wide range of genres, such as food, beverages, electronics, home furnishings, tools, pharmaceuticals, cosmetics, and pet products, but only reviews of products that could be bought and sold online. Therefore, the odour experiences captured in this study may not represent all aspects of daily life. However, these included not only positive but also negative evaluations of the various products mentioned above, so that a variety of olfactory experiences could be collected. In any case, this study proposes a novel approach to classifying the odours experienced by individuals in modern society.

During the selection of the top 100 odour nouns in this study, 27 inappropriate strings were manually excluded (S1 Table). In Japanese, the expression "no/noyouna/mitaina" + "nioi/kaori" is sometimes used with a word that does not necessarily represent the concrete object of the odour, such as "uniqueness" or "preference," which were excluded from our analysis. The combination of a noun and "nioi/kaori" is sometimes used to metaphorically describe an object that does not have a physical odour, such as "the odour of money" (the Japanese expression "kane no nioi"), meaning a feeling of making money, or "the odour of culture" (the Japanese expression "bunka no kaori"), meaning an atmosphere specific to a culture. Although we observed only a small number of occurrences of such words in our dataset—11 for "kane" and 5 for "bunka"—this may be because the dataset consisted of product reviews of concrete objects. If future studies encounter many instances of such nouns that do not represent the odour of concrete objects, it may be necessary to devise a system to filter them out. Currently, we are collecting data from social networking sites such as Twitter, using their APIs, in order to gather olfactory experience data from a wider range of situations. Such data can be utilized to conduct similar analyses in different countries and regions, and the resulting findings can be used to develop olfactory tests that are appropriate for each society and culture. By conducting this process longitudinally, we can monitor ever-changing daily odours over time.

## Supporting information

S1 Table The list of inappropriate strings as odour nouns. (PDF)
