# Correction: A new systematic collection and classification of odour words by using a product review dataset

**DOI:** 10.1371/journal.pone.0314583

**Published:** 2024-11-21

**Authors:** Naoya Zushi, Genki Takeuchi, Midori Ogawa, Naomi Gotow, Hideki Kakeya, Tatsu Kobayakawa, Saho Ayabe-Kanamura

The sixth author’s name is spelled incorrectly. The correct name is: Tatsu Kobayakawa. The correct citation is: Zushi N, Takeuchi G, Ogawa M, Gotow N, Kakeya H, Kobayakawa T, et al. (2023) A new systematic collection and classification of odour words by using a product review dataset. PLOS ONE 18(8): e0289368. https://doi.org/10.1371/journal.pone.0289368.
