# Supplementary material for: A new systematic collection and classification of odour words by using a product review dataset
Source: PLoS One. 2023 Aug 10;18(8):e0289368. doi: 10.1371/journal.pone.0289368 (PMC10414683; doi:10.1371/journal.pone.0289368)
Supplement: S1 Table — (PDF) [file pone.0289368.s001.pdf]

S1 Table. The list of inappropriate strings as odour nouns.

Excluding strings (Japanes	Excluding strings (Englis	Number of occurences
独特	uniqueness	27875
こ	*	21007
時	time	14147
特有	peculiar	12154
他	other	5335
とき	time	4866
好み	preference	4534
後	later	4008
商品	merchandise	3850
感じ	feeling	3516
お気に入り	favorite	3307
何	what	2628
そ	*	2572
開封時	when opening	2387
中	inside	2328
新品	brand new	2170
自体	itself	2020
こちら	this	1980
最初	first	1808
め	*	1777
上がり	*	1545
物	object	1327
瞬間	moment	1245
別	other	1120
製品	manufacture	1083
購入	purchase	903
自分	myself	901

*Blank columns are those that are not formed as nouns.
